# Comparative analysis of temporal trends of obesity and physical inactivity in Brazil and the USA (2011–2021)

**DOI:** 10.1186/s12889-023-17257-4

**Published:** 2023-12-14

**Authors:** Luciana Leite Silva Barboza, Américo Pierangeli Costa, Raphael Henrique de Oliveira Araujo, Ossian Guilherme Scaf Barbosa, João Luis Anwar El Sadat Paula Leitão, Mayda de Castro Silva, Guilherme Eckhardt Molina, Luiz Guilherme Grossi Porto

**Affiliations:** 1 Study Group in Physiology and Epidemiology of Exercise and Physical Activity (GEAFS), Postgraduate Program in Physical Education, University of Brasília (UnB), Campos Darcy Ribeiro, Brasília, DF 70910-900 Brazil; 2 Universidade Estadual de Londrina, Londrina, PR Brasil

**Keywords:** Lack of physical activity, Body weight, Epidemiology, Prevention and control, Public health

## Abstract

**Background:**

The prevalence of obesity is rising in all subregions of America, including Brazil. To understand the obesity problem in Brazil better, a possible approach could be to analyze its obesity trend by comparing it with the reality of a country that went previously through the epidemiological transition, such as the USA. In addition, the obesity trend must be analyzed in comparison with obesity risk factors trends, such as the physical inactivity (PI) trend. Our aim was comparatively to analyze the temporal trends of obesity between Brazil and the USA from the perspective of temporal trends of PI.

**Methods:**

We conducted a temporal trend study based on data from national cross-sectional surveys: the VIGITEL (Surveillance System for Factors of Health Risk and Protection for Chronic Diseases by Telephone Survey) for Brazil and the BRFSS (Behavioral Risk Factor Surveillance System) for the USA, comparing the annual prevalence of obesity and PI between 2011 and 2021. For the analysis of each temporal variation, linear regressions were performed with the Prais-Winsten test, and Pearson’s correlation coefficient was conducted to correlate the trends of the same variables between countries and of different variables within each country.

**Results:**

Considering the total sample, Brazil [coefficient (95%CI) 0.6 (0.4;0.7), *p* = 0.000] and the USA [coefficient (95%CI) 0.5 (0.5;0.6), *p* = 0.000] showed increasing trends in obesity. The tendency of PI was of stabilization in the two countries [Brazil: coefficient (95%CI) -0.03 (-0.3;0.2), *p* = 0.767 and USA coefficient (95%CI) -0.03 (-0.2;0.1), *p* = 0.584]. In addition, there was a correlation between obesity trends between Brazil and the USA (r = 0.971; *p* = 0.000), but there was no correlation between PI trends between the two countries, nor with obesity and PI trends within each country.

**Conclusions:**

In the last decade, there was a trend towards increasing obesity and stabilization in PI, both in Brazil and the USA. However, there was no association between temporal trends in obesity and physical inactivity in both countries. Our data reinforce a call to action to prevent and control obesity, going with and beyond PI reduction.

## Introduction

Obesity is a multifactorial disease, considered a risk factor for many chronic conditions, such as cardiovascular disease, type 2 diabetes, musculoskeletal disorders, and some types of cancer, in addition to being associated with psychological disorders and functional limitations [1]. Considered a severe public health problem, the prevalence of obesity is rising in all subregions of America, from 12.4% in men and 15.5% in women in the year 1980 to 34.4% in men and 36.2% in women in 2014. In southern Latin America, this increase within the same period was from 6.2% to 20.0% among men and from 11.5% to 26.4% among women [2]. In Brazil, a recent study evaluating socioeconomic inequality in obesity trend showed obesity prevalence estimates increase from 14.7% in 2007 to 20.0% in 2018 [3].

In addition to other factors, the fight against obesity is mainly related to the balance between food intake and energy expenditure. In this sense, physical activity is among the modifiable habits recommended to help control and prevent the disease [4]. Even without contributing significantly to the reduction of body weight, strategies promoting physical activity may have positive consequences for obesity control, mainly due to the decrease in cardiovascular risks [5]. In addition, the practice of physical activity is an essential factor for long-term weight loss and prevention of weight regain [6].

In Brazil, the prevalence of obesity has been increasing over the years, as demonstrated by time trend studies [3, 7, 8]. Among the solutions for combating obesity, encouraging the sufficient practice of physical activity has been wildly proposed [8]. However, only 30.1% of the Brazilian population aged 15 years or older practice sufficient leisure-time physical activity (150 min of moderate physical activity or 75 min of vigorous physical activity per week) [9].

High-income countries, such as the United States of America (USA), which have previously gone through an epidemiological transition resulting in changes in the population’s lifestyle [10], have been trying strategies to reduce obesity and the risk of other chronic diseases through public policies that encourage physical activity [11, 12]. Despite these initiatives, temporal trend studies have shown that the prevalence of obesity is still increasing in the USA, which is considered one of the countries with the highest proportion of obese people in the world [13].

To understand the obesity problem in Brazil better, which is one of the biggest middle-income countries in the world, a possible approach could be to analyze its obesity trend by comparing it with the reality of a country that has already undergone habit changes, such as the USA. In addition, the obesity trend must be analyzed in comparison with obesity risk factors trends, such as the physical inactivity trend, to understand the relationship between the two phenomena better. Therefore, public policies to address the obesity pandemic can be better directed.

Although studies on the temporal trend of obesity and physical inactivity already exist in isolation in both countries, no studies have compared the temporal trends of obesity between Brazil and the USA, nor simultaneously considering the temporal trend of physical inactivity. A more comprehensive analysis comparing both obesity and physical inactivity trends together and comparing the trends in Brazil along with the ones in the USA, which is a high-income country, one of the leaders in obesity prevalence and that underwent the epidemiological transition previously than Brazil, may generate new evidence to foster the obesity control and prevention in middle-income countries. Thus, our study aimed to comparatively analyze the temporal trends of obesity between Brazil and the USA in parallel with temporal trends of physical inactivity. As a specific aim, this analysis was also carried out from the stratification of the population by sex and age groups.

## Methods

We conducted a temporal trend study based on national cross-sectional surveys, comparing the annual prevalence of obesity and physical inactivity between 2011 and 2021 in Brazil and the USA. Data were obtained from national self-reported surveys: the VIGITEL (Surveillance System for Factors of Health Risk and Protection for Chronic Diseases by Telephone Survey) from Brazil and the BRFSS (Behavioral Risk Factor Surveillance System) from the USA. Both have similar methodologies: they are carried out annually and investigate risk and protective health factors in adults (≥ 18 years old) through telephone interviews. VIGITEL is carried out by the Brazilian Ministry of Health, started in 2006, and collects data from individuals in the 26 Brazilian state capitals plus the Federal District. VIGITEL’s sampling is carried out in two stages; the first consists of drawing at least 10,000 telephone lines in each city and the second of drawing an adult residing in the selected household [14]. The BRFSS, which has existed since 1984, is carried out by the CDC (Centers for Disease Control and Prevention), and in 2021 it collected data from individuals in all 50 states and four American territories. The first stage of sampling consists of a telephone draw. Afterward, the drawn telephone numbers are divided into two groups, high-density, and low-density strata, with each stratum having the same chance of being drawn. In the last stage, a household resident is selected to answer the questionnaire [15]. Table 1 presents the total sample size in each country, of individuals who answered questions about obesity and physical inactivity, to calculate the prevalence of these variables, according to the years.

**Table 1:** Total sample size used to calculate the prevalence of obesity and physical inactivity, according to country and year

Year	Brazil	USA	
	VIGITEL To Obesity/Physical inactivity	BRFSS To Obesity	BRFSS To Physical inactivity
	n	n	n
2011	54,144	470,700	475,078
2012	45,448	442,230	465,777
2013	52,929	457,487	450,093
2014	40,853	425,875	454,431
2015	54,174	398,316	396,649
2016	53,210	438,479	476,876
2017	53,034	408,448	410,595
2018	52,395	396,022	430,259
2019	52,443	374,073	388,044
2020	27,077	353,841	394,153
2021	27,093	385,204	430,714

Note: VIGITEL = Surveillance System for Factors of Health Risk and Protection for Chronic Diseases by Telephone Survey; BRFSS = Behavioral Risk Factor Surveillance System. VIGITEL data refer to the total sample size for calculating the prevalence of both obesity and physical inactivity

### Variables

For the classification of obesity, both in VIGITEL and BRFSS, self-reported measures of weight and height were used, considering Body Mass Index (BMI) values ≥ 30 kg/m^2^. Physical inactivity in VIGITEL is defined as when an individual did not accumulate any physical activity during leisure-time in the last three months; did not make intense physical efforts at work, did not go to work or course/school walking or cycling for a minimum of 20 min round trip and was not responsible for the heavy cleaning of their house. BRFSS considers the absence of any PA in leisure-time in the last month in addition to regular work through the following question: “During the past month, other than your regular job, did you participate in any physical activities or exercises such as running, calisthenics, golf, gardening, or walking for exercise?“. Studies carried out in Brazil [16, 17] and the USA [18, 19] tested the validity and reliability of the questionnaires used respectively in VIGITEL and BRFSS to assess physical activity. Obesity and physical inactivity data were extracted from official country reports [20, 21]. For the present study, we considered the prevalence for the total population of adults and also stratified by sex and age groups (18 to 24 years; 25 to 34 years; 35 to 44 years; 45 to 54 years; 55 to 64 years old and 65+), which were similar between both reports.

### Statistical analysis

In the analyses, the prevalence for each year is presented in percentage, considering the sample weight according to the methodology of each country. The total difference (2021 minus 2011) between the last and first years was calculated in percentual points (p.p.). For the analysis of each temporal variation, linear regressions were performed with the Prais-Winsten test, using the autocorrelation option, to verify trends of increase/decrease with significant values (*p* < 0.05) or stability with non-significant values (*p* ≥ 0.05) [22]. Pearson’s correlation coefficient was conducted to compare the trends of the same variables between countries and of the different variables within each country. Analyzes were performed using Stata software, version 15.0.

## Results

**Fig. 1:**
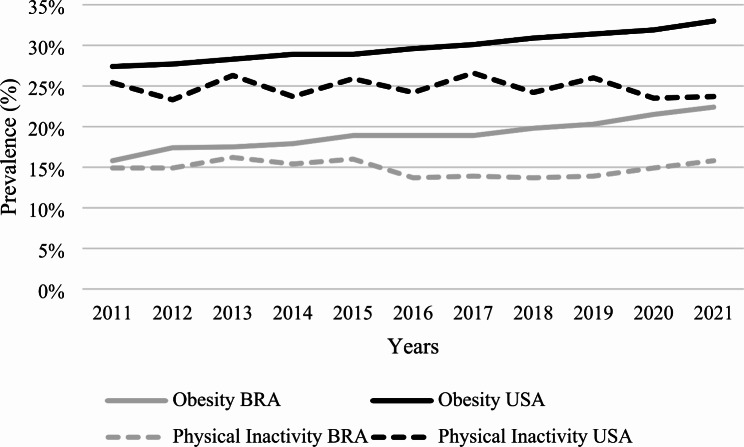
Temporal trends of obesity and physical inactivity in Brazil and the USA (2011 to 2021)

Figure 1 graphically shows the temporal trend of obesity and physical inactivity in the two countries. Despite the differences in absolute prevalence values, the trends of the analyzed variables behaved similarly in both countries. Notably, the USA prevalence values were, on average, 10.8 p.p. and 10.0 p.p. above Brazil in obesity and physical inactivity, respectively. Notably, the obesity curves were very similar in both countries. We observed a more significant oscillation in the physical inactivity curve in the USA during the analysis period, but with similar values when considering the start and the endpoints of analysis (2011–2021).

Table 2 presents the correlation values between the analyzed variables. Correlation is observed only between the temporal trends of obesity in Brazil and the USA. There was no correlation between the obesity and physical inactivity variables within each country, as well as there was no correlation between the physical inactivity variables in the two countries.

**Table 2:** Correlation between temporal trends of obesity and physical inactivity in Brazil and the USA (2011 to 2021)

Variables	Obesity BRA	Physical Inactivity BRA	Obesity USA	Physical Inactivity USA
Obesity BRA	---			
Physical Inactivity BRA	r = -0.088 *p* = 0.797	---		
Obesity USA	**r = 0.971** ***p = 0.000***		---	
Physical Inactivity USA		r = 0.005 *p* = 0.988	r = -0.216 *p* = 0.523	---

Note: r = Pearson’s correlation. *p* = level of significance of 5%

Table 3 presents the prevalences and analyses of the temporal trends of obesity and physical inactivity in Brazil, while in Table 4, we have the results from the USA. As shown in Table 3, between 2011 and 2021, obesity in Brazil showed an increasing trend, regardless of sex and age group. On the other hand, physical inactivity in Brazil led to a tendency towards stability, except for a downward trend among individuals aged 45 to 54. In the USA (Table 4), the tendency of obesity was to increase in all the stratifications groups, while the trend of physical inactivity was of stability, except between the first and the last age categories, which presented a tendency of increase among the younger individuals and a decrease among older ones. In other words, Brazilian obesity data showed a tendency to increase in both sexes and all age groups. We observed the same tendency in the USA. As for the physical inactivity prevalence, Brazil showed stability in both sexes and 83.3% of all age groups. The USA also showed stability in both sexes and 66.7% of the age groups.

**Table 3:** Prevalence (%) and temporal trend analysis of obesity and physical inactivity in adults, by sex and age group, according to VIGITEL (Brazil) data from 2011 to 2021

Variable	Stratification	2011	2012	2013	2014	2015	2016	2017	2018	2019	2020	2021	2021 − 2011	Coefficient	95%CI	*p*-value	Tendency
Obesity	Total	15.8	17.4	17.5	17.9	18.9	18.9	18.9	19.8	20.3	21.5	22.4	6.6	0.6	0.4;0.7	0.000	↑
	Male	15.6	16.5	17.5	17.6	18.1	18.1	19.2	18.7	19.5	20.3	22.0	6.4	0.5	0.4;0.6	0.000	↑
	Female	16.0	17.3	17.5	18.2	19.7	19.6	18.7	20.7	21.0	22.6	22.6	6.6	0.6	0.5;0.7	0.000	↑
	18 to 24 y	6.6	7.5	6.3	8.5	8.3	8.5	9.2	7.4	8.7	9.9	12.2	5.6	0.4	0.2;0.6	0.003	↑
	25 to 34 y	14.8	15.1	15.0	15.1	17.9	17.1	16.5	18.0	19.3	19.6	20.8	6.0	0.6	0.4;0.7	0.000	↑
	35 to 44 y	18.9	19.7	20.1	22.0	23.6	22.5	22.3	23.2	22.8	24.7	25.5	6.6	0.6	0.3;0.8	0.000	↑
	45 to 54 y	21.7	22.6	22.5	21.3	21.7	22.8	23.3	24.0	24.5	27.1	26.2	4.5	0.5	0.2;0.7	0.004	↑
	55 to 64 y	20.4	23.4	24.4	23.1	22.7	22.9	22.6	24.6	24.3	26.2	26.2	5.8	0.4	0.1;0.7	0.009	↑
	65 y or more	17.7	19.0	20.2	19.8	19.4	20.3	20.3	21.5	20.9	20.2	21.8	4.1	0.3	0.1;0.4	0.001	↑
Physical Inactivity	Total	14.9	14.9	16.2	15.4	16.0	13.7	13.9	13.7	13.9	14.9	15.8	0.9	-0.03	-0.3;0.2	0.767	—
	Male	15.1	15.2	16.8	16.2	16.0	12.2	13.9	13.0	13.8	14.1	15.6	0.5	-0.1	-0.5;0.2	0.360	—
	Female	14.7	14.6	15.7	14.7	16.0	14.9	13.9	14.2	14.0	15.5	16.0	1.3	0.03	-0.2;0.2	0.735	—
	18 to 24 y	13.3	12.6	13.7	12.0	15.1	12.4	14.5	12.5	12.9	14.5	10.7	-2.6	-0.02	-0.1;0.1	0.746	—
	25 to 34 y	11.2	10.6	11.6	12.3	11.9	9.4	8.9	9.7	10.8	11.7	13.0	1.8	0.1	-0.3;0.5	0.590	—
	35 to 44 y	11.2	11.8	12.4	10.7	11.5	9.3	9.3	9.7	10.9	10.7	12.2	1.0	-0.03	-0.3;0.3	0.814	—
	45 to 54 y	13.4	12.8	13.7	13.9	12.9	10.7	11.6	10.8	10.4	11.6	11.8	-1.6	-0.2	-0.5;-0.01	0.038	↓
	55 to 64 y	18.9	16.9	20.2	15.9	18.2	15.2	14.4	15.4	14.6	16.2	17.9	-1.0	-0.3	-0.6;0.05	0.092	—
	65 y or more	32.0	35.8	38.4	38.2	37.5	36.1	35.2	33.2	31.8	32.8	37.6	5.6	0.2	-0.6;1.1	0.574	—

Note: VIGITEL = Surveillance System for Factors of Health Risk and Protection for Chronic Diseases by Telephone Survey; Y = Years; 2021 − 2011 = total difference between 2021 and 2011 in percentual points; Coefficient = Prais-Winsten regression coefficient; 95%CI = Confidence Interval 95%; *p*-value = level of significance of 5%; ↑ = tendency of increase; ↓ = tendency of decrease; — = tendency of stability. Prevalences from 2011 to 2021 are presented in percentages

**Table 4:** Prevalence (%) and temporal trend analysis of obesity and physical inactivity in adults, by sex and age group, according to BRFSS (USA) data from 2011 to 2021

Variable	Stratification	2011	2012	2013	2014	2015	2016	2017	2018	2019	2020	2021	2021 − 2011	Coefficient	95%CI	*p*-value	Tendency
Obesity	Total	27.4	27.7	28.3	28.9	28.9	29.6	30.1	30.9	31.4	31.9	33.0	5.6	0.5	0.5;0.6	0.000	↑
	Male	27.8	28.0	28.3	29.0	29.1	29.6	30.2	30.6	30.6	31.7	32.3	4.5	0.4	0.4;0.5	0.000	↑
	Female	27.1	27.4	28.3	28.8	28.6	29.5	30.0	31.3	32.1	32.1	33.7	6.6	0.6	0.5;0.7	0.000	↑
	18 to 24 y	15.2	15.0	15.4	15.9	16.7	17.3	16.5	18.1	18.9	19.5	20.7	5.5	0.5	0.4;0.7	0.000	↑
	25 to 34 y	25.9	25.6	26.4	27.0	26.7	27.2	28.2	29.5	29.5	30.9	32.0	6.1	0.6	0.4;0.8	0.000	↑
	35 to 44 y	29.9	31.3	31.7	32.1	32.1	33.1	33.0	34.5	34.6	35.5	36.8	6.9	0.6	0.5;0.7	0.000	↑
	45 to 54 y	32.6	32.4	33.3	33.7	34.0	35.1	35.9	36.9	37.6	38.1	39.3	6.7	0.7	0.6;0.8	0.000	↑
	55 to 64 y	32.6	33.3	33.5	34.2	33.4	34.2	35.4	35.1	36.0	36.3	38.1	5.5	0.5	0.3;0.6	0.000	↑
	65 y or more	25.3	25.8	26.5	27.5	27.6	28.0	28.5	28.9	29.3	29.3	29.5	4.2	0.4	0.3;0.5	0.000	↑
Physical Inactivity	Total	25.4	23.3	26.3	23.7	25.9	24.2	26.6	24.2	26.0	23.5	23.7	-1.7	-0.03	-0.2;0.1	0.584	—
	Male	23.9	21.2	24.5	21.7	24.6	21.9	25.0	21.7	24.4	21.5	21.1	-2.8	-0.1	-0.2;0.09	0.339	—
	Female	26.9	25.3	27.9	25.6	27.0	26.4	28.1	26.6	27.5	25.4	26.1	-0.8	0	-0.1;0.1	0.972	—
	18 to 24 y	16.9	14.7	18.3	16.2	17.4	15.4	18.4	16.1	19.8	17.5	17.0	0.1	0.2	0.05;0.3	0.014	↑
	25 to 34 y	22.1	19.0	22.5	19.5	21.8	19.2	22.6	19.1	22.7	18.8	19.2	-2.9	-0.09	-0.2;0.05	0.184	—
	35 to 44 y	24.3	21.4	24.9	21.4	25.5	21.7	25.8	21.9	24.0	21.0	20.5	-3.8	-0.1	-0.4;0.06	0.141	—
	45 to 54 y	26.1	23.9	27.3	24.5	27.6	25.0	27.8	25.1	27.1	23.5	23.6	-2.5	-0.1	-0.3;0.1	0.394	—
	55 to 64 y	28.1	26.7	28.7	26.7	28.3	28.3	29.5	27.4	28.5	26.0	26.7	-1.4	-0.06	-0.3;0.1	0.499	—
	65 y or more	33.0	31.5	33.1	31.2	31.3	32.2	32.1	31.9	31.0	30.6	31.0	-2.0	-0.1	-0.3;-0.05	0.010	↓

Note: BRFSS = Behavioral Risk Factor Surveillance System; Y = Years; 2021 − 2011 = total difference between 2021 and 2011 in percentual points; Coefficient = Prais-Winsten regression coefficient; 95%CI = Confidence Interval 95%; *p*-value = level of significance of 5%; ↑ = tendency of increase; ↓ = tendency of decrease; — = tendency of stability. Prevalences from 2011 to 2021 are presented in percentages

## Discussion

In our study, we have comparatively analyzed the temporal trends of obesity in Brazil and the USA in parallel with the temporal analysis of physical inactivity in these countries. To the best of our knowledge, this is the first study analyzing temporal trends of obesity between Brazil and the USA and simultaneously considering its association with the temporal trend of physical inactivity. The main findings showed a different tendency of temporal trends between obesity and physical inactivity if we compare the two variables within each country. While obesity tended to increase over time, physical inactivity tended to stabilize between 2011 and 2021. Comparing the two countries, we found a correlation between temporal trends of obesity. The obesity prevalence trend in Brazil in the last decade was almost identical to the USA, noting that the current difference of about 11 p.p. already existed at the beginning of the period. Also, despite a more significant oscillation of the prevalence curve of physical inactivity in the USA as compared to Brazil, during the last decade, in both countries, the trend of physical inactivity showed no association with the trends of obesity. In other words, the tendency of stability in physical inactivity prevalence estimates was not associated with the tendency of obesity, which increased in both countries in the same period.

Our results contradict previous studies analyzed in a recent systematic review demonstrating an association between obesity and physical inactivity [23]. Also, relatively old British epidemiological data suggest that low levels of physical activity may play an essential role in obesity [24]. However, apart from this British study, none of these associations reported in the above-mentioned review study considered the temporal trend analysis. A possible explanation for obesity showing a growing trend, even with a trend towards stability in physical inactivity, is the multifactorial determinants of obesity and the fact that other factors may have a more substantial impact on obesity than physical inactivity alone, such as sedentary behavior, diet, genetics, sleep patterns [25], and mental health [26].

In Brazil, a national study carried out in 2019 revealed that 30,1% of adults spent 6 or more hours per day in screen-based sedentary behaviors like watching TV and using computer or other screens [27]. Over a period of 10 years, between 2008 and 2018, it was observed an increase in the percentage of energy intake from ultra-processed foods and a decrease from plant-based natural/minimally processed foods in Brazilian adults [28]. Similar results were found among US adults, in which a study with accelerometers revealed median time of 8 h per day in sedentary behaviors [29]. Also, there is an increase in consumption of ultra-processed foods, which has been rising in the last two decades [30].

Although sedentary behavior is a phenomenon interrelated to physical activity, both are conceptually different [31] and can manifest independently, with the individual being able to reach the physical activity recommended by the WHO criterion while presenting high levels of sedentary behavior [32]. Notably, there is still much to understand about the independent detrimental effect of sedentary behavior on cardiometabolic diseases, such as obesity. At the same time, physical activity might be an effect modifier of the association between sedentary behavior and cardiometabolic health outcomes, making the interpretation of obesity trend analyses even more complex [33]. After that, our data do not support the understanding that physical activity does not affect populational obesity prevalence, which would imply the denial of solid evidence-based public health recommendations. However, our findings do support the interpretation that public policies or health programs aimed at controlling obesity should not be exclusively based on the promotion of physical activity.

Despite the finding that other factors may be more associated with the increase in obesity, the result of the stabilization trend of physical inactivity in both countries reveals a need to increase efforts so that this trend is reduced [34]. In addition, considering a different approach to our results, we cannot estimate the impact of the obesity trend in both countries if the physical inactivity trend had increased between 2011 and 2021. However, based on current evidence, it is reasonable to admit that if physical inactivity had experienced an increased trend in the analyzed period, the obesity prevalence would have probably been even higher [23]. In other words, decreasing the trend of physical inactivity could potentially help to reduce or stabilize the increasing trend of obesity.

The growing trend of obesity in both countries had already been confirmed in previous studies carried out individually in both countries [7, 8, 13], but our study found that these trends are correlated. In this sense, actions need to be taken in Brazil so that the prevalence of obesity does not reach values as high as those currently presented in the USA. Our findings are likely generalizable to other middle-income countries, similar to Brazil. Suppose no action is taken, obesity will continue to increase, mainly in low- and middle-income countries. In that case, the World Obesity Atlas projects a prevalence of 33% for women and 26% for men in Brazil, placing the country among the 11 with more women and 9 with more men with obesity by 2030 [35].

Research analyzing temporal trends of risk and protective behaviors related to chronic diseases is essential for understanding certain phenomena and predicting population behaviors that may be encouraged or discouraged to improve people’s health. Comparing temporal trends from two different contexts is also essential, especially when one of them, the USA, is the biggest economy in the world, in which we observe one of the highest prevalence estimates of obesity. Importantly, our data demonstrate that the Brazilian obesity trend in the last decade is very similar to one of the leading countries in obesity prevalence. This concerning finding imposes, per se, an urgent and collective call to action aiming to revert this scenario.

Of note, our findings must not be a disincentive to physical activity promotion. Apart from all the health benefits associated with an active lifestyle [36], our results suggest that physical inactivity alone could not explain the obesity trend in both countries. On the other side, the stability trend of the high prevalence of physical inactivity in both countries demonstrates the need to create public policies that consider physical activity as an essential component in the fight against obesity and other chronic diseases, together with other factors such as healthy eating and reducing sedentary behavior (potentials). In this way, initiatives to promote physical activity implemented in Brazil, such the Health Academy Program (*Programa Academia da Saúde*) [37], and Healthy People 2030 [38], in the USA, could include specific aims focusing on reducing obesity through physical activity. Beside that, cities need to increase the opportunities for physical activity practices that are also attractive to obese people, considering their limitations and needs [39, 40].

This study has some limitations. VIGITEL uses representative data from the capitals of the Brazilian states, disregarding the prevalence of the interior, which has its particularities. Because of this, the sample size in the USA is larger than in Brazil. However, due to the similarity of the data collection methods, and because the sampling from both countries are representative of the target population of each country, both surveys are comparable. Also, an intrinsic limitation in comparing health indicators between central and peripheral economies could be raised. However, we did not compare causal relationships or associated factors frequently modified by economic factors. We aimed to reflect on the Brazilian reality based on a comparison with a leading country in obesity worldwide, which previously went through an epidemiological transition. Another limitation is that the definition of physical inactivity in Brazil considers the absence of physical activity in the last three months in leisure-time, work, transport, and domestic domains. In contrast, the USA considers the absence of physical activity only in the last month in the leisure-time domain. As differences remain across years, trend comparison minimizes cross-country differences. Another limitation that might be pointed out is that many other factors besides physical inactivity, especially diet, stress, sleep disorders, and sedentary behavior, interfere with obesity. However, the impact of different obesity-associated factors is beyond the scope of the study, and physical inactivity was analyzed in isolation. The causes of obesity, both individual and socioeconomic, were also beyond our aims. Furthermore, the present study did not consider other socioeconomic factors that could explain differences mainly between the two countries.

## Conclusion

In the last decade, there was a trend to increase in obesity and stabilization in physical inactivity, both in Brazil and the USA. There was no association between temporal trends in obesity and physical inactivity in both countries. Despite a lower obesity prevalence in Brazil, its growth rate in the last ten years is similar to that observed in the USA, one of the world’s leading countries in obesity prevalence. Our data reinforce the need for a call to action to prevent better and control obesity, going with and beyond physical inactivity reduction.

## Data Availability

Data from the VIGITEL (Surveillance System for Factors of Health Risk and Protection for Chronic Diseases by Telephone Survey) is available on the Brazilian Ministry of Health website: https://www.gov.br/saude/pt-br/centrais-de-conteudo/publicacoes/publicacoes-svs/vigitel. And the BRFSS (Behavioral Risk Factor Surveillance System) in the CDC (Center of Disease Control and Prevention website: https://nccd.cdc.gov/dnpao_dtm/rdPage.aspx?rdReport=DNPAO_DTM.ExploreByTopic&islClass=OWS&islTopic=&go=GO.
